# Characterization of mineral concentrations and yields from colostrum to mature milk of multiparous Holstein cattle

**DOI:** 10.3168/jdsc.2026-1057

**Published:** 2026-05-20

**Authors:** K. Klein, D.J. Seymour, L.E. Engelking, A.J. Fischer-Tlustos, J.B. Daniel, J.N. Wilms, J. Martín-Tereso, M.A. Steele

**Affiliations:** 1 Department of Animal Biosciences, Animal Science and Nutrition, University of Guelph, Guelph, ON, Canada N1G 1Y2; 2 Trouw Nutrition R&D, 5830 AE Boxmeer, the Netherlands; 3 Department of Agricultural, Food and Nutritional Science, University of Alberta, Edmonton, AB T6G 2P5

## Abstract

Colostrum and transition milk contain a variety of bioactive components that promote the health and development of neonatal calves. This work characterizes how mineral composition and yields evolve from colostrum to transition and mature milk. Milk samples were collected from 15 multiparous Holstein dairy cattle at milkings 1 through 6, 8, 10, 12, and 14. Macromineral (Ca, Mg, P, Na, K, S) concentrations were determined by inductively coupled plasma–optical emission spectrometry, with the exception of Cl, which was analyzed by potentiometric titration, whereas trace mineral (Zn, Cu, Mn, Fe, Co, Se, Mo) concentrations were determined by inductively coupled plasma–mass spectrometry. The concentrations (mass per kg) of all minerals were highest in colostrum and progressively decreased from transition to mature milk, with the exceptions of K, Cl, Mn, and Mo, which maintained similar concentrations in both transition and mature milk. However, trends in mineral yields largely reflected increasing milk volume, with osmotically relevant macrominerals increasing (Na, K, Cl) with volume, or linked to somatic growth (Ca) to increase across lactation, whereas trace minerals declined (Co) or increased (Cu) throughout lactation. These results demonstrate that individual minerals follow a specific temporal pattern across lactation in connection with their function, and thus the stage of lactation should be considered when evaluating milk mineral composition.

## Graphical Abstract

**Figure:**
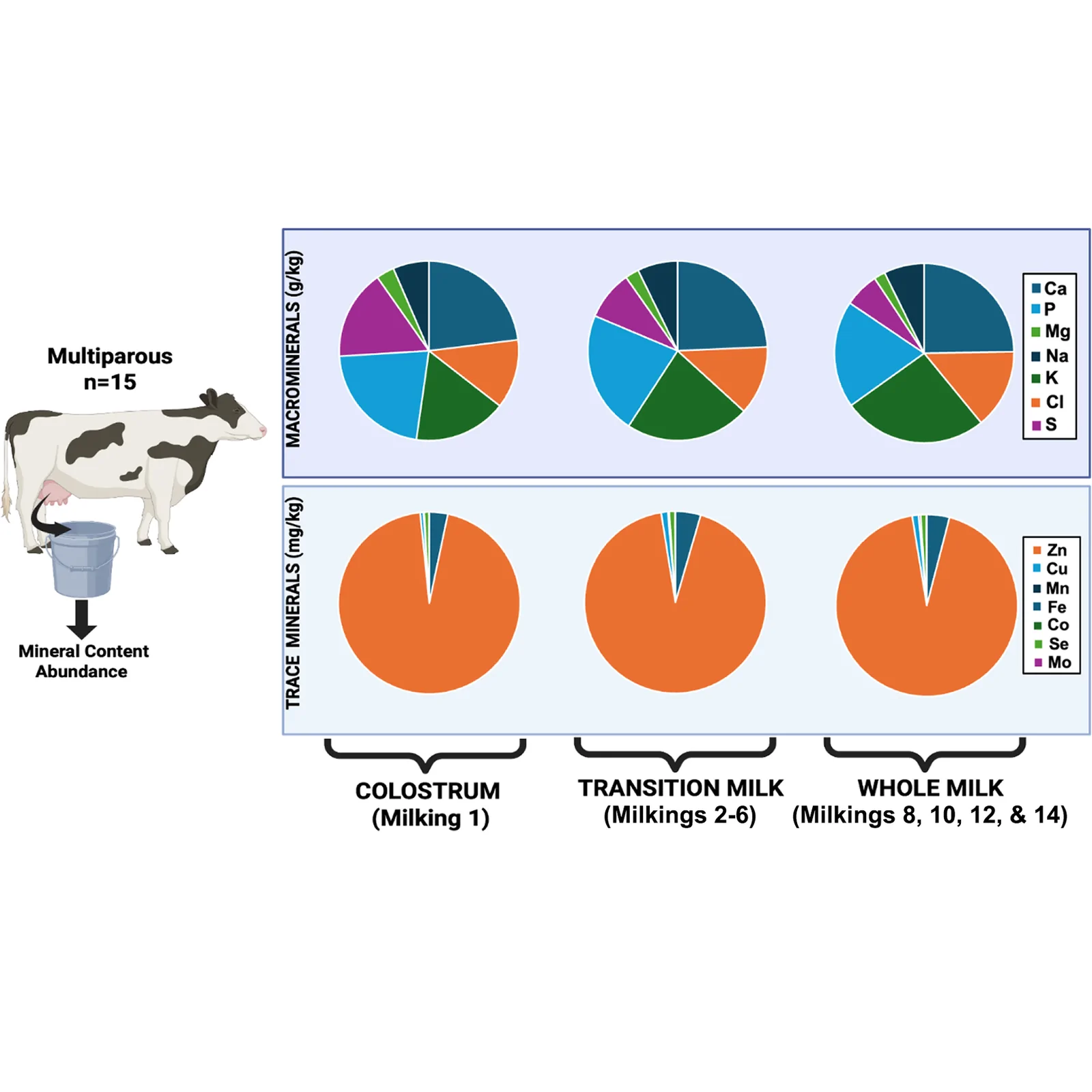

Colostrum, defined as the first milk collected after calving, is vital for calf health, growth, and development (Godden et al., 2019). In addition to feeding a high-quality (>50 g of IgG per liter) colostrum meal after birth, recent literature has also highlighted the benefits of feeding transition milk, defined as milkings 2 through 6, which has been associated with improved calf growth and health outcomes (Hare et al., 2020; Van Soest et al., 2020), likely attributed to its unique composition relative to mature milk (Renaud and Steele, 2025) in connection with the calf's age-specific developmental requirements. The temporal changes of colostrum to transition milk during the shift from colostrogenesis to established lactation may provide insight into neonatal nutritional requirements and associated biological signaling during early life. Previous studies have addressed key changes in early-lactation milk composition and have largely focused on fat (Wilms et al., 2024), protein (Nissen et al., 2017; Fahey et al., 2020), and immunoglobulins (Ahmann et al., 2021; Costa et al., 2021). However, changes in many other components, such as minerals, have not yet been thoroughly investigated.

Minerals are integral to nearly every physiological function in the body (Underwood and Suttle, 1999). In neonatal animals, elemental nutrients are crucial building blocks for rapid postnatal growth and tissue development and play a role in metabolic adaptations to extrauterine life (Land and Schoenau, 2008). We will refer to Ca, P, Mg, Na, K, Cl, and S as macrominerals, as they are present in biological systems, particularly in animals, in greater amounts than other elemental nutrients. Despite serving multiple roles, they can be broadly classified according to their most quantitatively relevant functions. These include structural roles as skeletal building blocks (Ca, P), components of amino acids (S), and physiological roles as monovalent or divalent electrolytes involved in osmotic balance, acid-base regulation, membrane permeability, and cellular signaling (Ca, Mg, Na, K, Cl; Hunt and Nielsen, 2009). The less-abundant trace minerals (Zn, Cu, Mn, Fe, Co, Se, Mo) typically support metabolic functions as metal groups in enzymes and other proteins (Zn, Cu, Mn, Fe, Mo), as part of the essential amino acid selenomethionine coded in highly functional proteins (Se), or as a unique component of vitamin B_12_ (Co; Hunt and Nielsen, 2009). Although individual minerals often fulfill multiple physiological roles, all are fundamental to directly or indirectly contributing to the broader roles of nutrient transfer, metabolic regulation, and biological signaling.

Milk mineral supply of both macro- and trace minerals supports neonatal calf development after birth through roles in growth, immune function, metabolic regulation, hydration, acid-base balance, and enzymatic and hormonal activity (Underwood and Suttle, 1999). From a practical perspective, understanding mineral composition throughout the course of lactation can serve as a biological reference for the formulation of milk replacers and feeding strategies intended to better approximate the functions of maternal milk during early life. Despite the recognized importance of minerals to calf health and growth (Olson, 2007; Osorio et al., 2012), the mineral fractions of colostrum and transition milk have not been well characterized in cattle. Therefore, the objective of this study was to describe the temporal changes in mineral compositions of colostrum, transition milk, and mature milk in Holstein cows.

All procedures were approved by the University of Alberta Animal Care and Use Committee for Livestock (AUP no. 00002015) in accordance with guidelines set by the Canadian Council on Animal Care. This study was conducted at a 500-head commercial freestall dairy in Alberta, Canada, between May and June 2017. Fifteen multiparous Holstein dairy cattle were randomly selected from the herd for use in this study based on parity and calving date. Details on general animal husbandry and experimental conditions were previously reported (Fahey et al., 2020; Fischer-Tlustos et al., 2020). In brief, cattle were group-housed and fed a common barley silage–based dry cow ration before calving, after which they were switched to a lactating ration composed of barley and corn silage (Fischer-Tlustos et al., 2020). Cattle had ad libitum access to water via watering troughs. At the onset of lactation, cattle were milked twice daily at ∼0500 and 1600 h. For the duration of the study, no cattle exhibited any clinical symptoms of illness or received antibiotics.

Samples of colostrum were collected at the first milking following calving (4.3 ± 0.6 h postcalving). Homogeneous samples of colostrum and milk were collected directly from the milking equipment (BouMatic, Madison, WI) into 50-mL Falcon tubes at each milking for the first 6 milkings, and at milkings 8, 10, 12, and 14, where milkings corresponded to the sequential milking number for each cow. An aliquot of ∼20 mL was taken from each milk sample and stored at −20°C for analysis of mineral concentration. To determine the mineral concentrations of colostrum and milk, samples were thawed and freeze-dried before shipment to the Agriculture and Food Laboratory (University of Guelph, Guelph, ON, Canada). Macrominerals, except for Cl, were quantified by microwave digestion and inductively coupled plasma–optical emission spectroscopy (**ICP-OES**; method 2011.14, AOAC International, 2023), whereas trace minerals were quantified by inductively coupled plasma–mass spectrometry (**ICP-MS**; method 6020, EPA, 1994). Chloride was quantified separately at MasterLab (Trouw Nutrition, Saint-Hyacinthe, QC, Canada) via potentiometric titration. Osmolality was measured by freezing-point depression using an osmometer (3300 Micro Osmometer, Advance Instruments, Norwood, MA) following the manufacturer's instructions. Due to limitations in sample availability, Cl was not measured for 11 out of 189 samples across different milkings.

All data were analyzed using SAS v9.4 (SAS Institute Inc., Cary, NC). Analyses of variance were conducted using the GLIMMIX procedure, where milking was considered a fixed effect and animal was considered a random effect. Repeated measures on animals were modeled using a spatial power covariance structure. Plots of conditional studentized residuals were evaluated to determine whether data were normally distributed; if data were not normally distributed, models were re-fit using a log-normal distribution and re-evaluated. It was determined that the concentrations of S, Mn, Fe, Co, and Se and the yields of S, Zn, Fe, and Mn followed log-normal distribution. For all analyses, the Kenward–Roger correction was used to adjust the denominator degrees of freedom. A Newton–Raphson optimization with ridging was requested using the NLOPTIONS statement, allowing for 100 iterations to reach model convergence. *P*-values for multiple means comparisons were adjusted using the Tukey-Kramer method. Preplanned contrasts comparing colostrum (milking 1), transition milk (milkings 2–6), and mature milk (milkings 8–14) were performed. Tests were considered statistically significant where *P* < 0.05 and trends where 0.05 ≤ *P* < 0.10.

As reviewed by McGrath et al. (2016) and Puppel et al. (2019), there is a lack of consensus regarding the mineral concentrations of colostrum and transition milk; this is further amplified by the lack of peer-reviewed studies in this area. The results presented herein demonstrate that concentrations of all minerals were the highest in colostrum and decreased from transition to mature milk, whereas mineral yields showed more variable responses driven by rapid increases in milk volume following parturition (Table 1, Table 2).

**Table 1:** Milk mineral yields in early lactation of multiparous Holstein dairy cows (n = 15) milked twice daily; values represent means with 95% CI in parentheses for log-normal data

Mineral	Milking number										SED^2^	*P*-value^1^			
	1	2	3	4	5	6	8	10	12	14		Milking number	CvT	CvM	TvM
Ca (g)	17.9^a^	20.1^ab^	23.6^ab^	24.4^ab^	23.9^ab^	23.7^ab^	26.5^ab^	26.9^ab^	28.0^ab^	29.6^b^	3.46	0.067	0.052	<0.001	0.013
P (g)	17.3	19.1	22.8	22.4	20.7	19.7	21.9	21.3	22.1	22.6	3.13	0.76	0.13	0.060	0.52
Mg (g)	2.66	2.35	2.47	2.42	2.21	2.21	2.25	2.18	2.25	2.30	0.396	0.98	0.28	0.18	0.67
Na (g)	5.23	5.39	6.12	7.34	7.53	7.62	8.16	7.84	7.95	7.98	1.256	<0.001	0.059	0.001	0.001
K (g)	13.1^a^	17.7^ab^	21.3abc	23.3abc	24.2abc	24.0abc	28.2^bc^	27.8^bc^	31.1^c^	31.3^c^	4.41	<0.001	0.002	<0.001	<0.001
Cl (g)	11.7abc	8.93^a^	9.81^ab^	12.9abc	14.5abc	14.3abc	14.8abc	15.1abc	15.5^bc^	17.9^c^	2.17	0.012	0.80	0.022	<0.001
S (g)	10.0	9.42	8.46	7.26	6.93	6.23	5.65	6.31	6.64	6.71		0.43	0.11	0.014	0.16
	(7.05–14.3)	(6.64–13.4)	(6.01–11.9)	(5.11–10.3)	(4.93–9.76)	(4.50–8.63)	(4.08–7.82)	(4.51–8.83)	(4.74–9.29)	(4.68–9.62)					
Zn (mg)	148	112	105	98.9	97.0	89.1	79.0	81.3	87.5	95.4		0.65	0.044	0.018	0.35
	(98.9–221)	(75.5–167)	(70.9–155)	(66.3–147)	(65.6–143)	(61.1–130)	(54.2–115)	(55.1–120)	(59.2–129)	(62.9–145)					
Cu (mg)	0.93^a^	1.27^ab^	1.63^ab^	1.69^b^	1.29^ab^	1.14^ab^	1.01^ab^	1.07^ab^	1.12^ab^	1.27^ab^	0.224	0.021	0.006	0.27	0.011
Mn (μg)	112	107	89.4	74.0	59.8	61.5	69.6	83.9	86.6	94.7		0.52	0.060	0.10	0.61
	(71.4–174)	(69.1–167)	(57.9–138)	(47.5–115)	(38.7–92.3)	(40.4–93.6)	(45.7–106)	(54.5–129)	(56.1–133)	(59.9–149)					
Fe (mg)	5.34	5.41	6.15	5.12	4.86	4.26	3.79	3.93	3.62	3.98		0.73	0.83	0.12	0.070
	(3.63–7.87)	(3.69–7.92)	(4.23–8.96)	(3.49–7.52)	(3.34–7.07)	(2.97–6.12)	(2.64–5.42)	(2.71–5.69)	(2.49–5.25)	(2.66–5.92)					
Co (μg)	22.6^a^	19.7^ab^	16.7^ab^	13.1^ab^	11.4^b^	10.2^b^	9.60^b^	8.78^b^	9.17^b^	9.40^b^	3.86	0.032	<0.001	<0.001	0.051
Se (mg)	1.62	1.54	1.25	1.29	1.15	1.05	1.12	1.13	1.17	1.11	0.258	0.11	0.045	0.009	0.22
Mo (μg)	294	262	299	290	351	329	386	405	390	380	56.3	0.28	0.77	0.036	0.014

a–c Means with different letters within rows are significantly different (*P* < 0.05).

1 CvT = colostrum (milking 1) vs. transition milk (milkings 2–6); CvM = colostrum (milking 1) vs. mature milk (milkings 8–14); TvM = transition (milkings 2–6) vs. mature milk (milkings 8–14).

2 Standard error of the difference. Values excluded for log-normal data.

**Table 2:** Milk mineral concentrations and osmolality in early lactation of multiparous Holstein dairy cows (n = 15) milked twice daily; values represent means with 95% CI in parentheses for log-normal data

Item	Milking number										SED^2^	*P*-value^1^			
	1	2	3	4	5	6	8	10	12	14		Milking number	CvT	CvM	TvM
Ca (g/kg)	3.14^a^	2.44^b^	2.15^bc^	2.07^bc^	1.90^c^	1.78^c^	1.90^c^	1.82^c^	1.77^c^	1.92^c^	0.18	<0.001	<0.001	<0.001	0.001
P (g/kg)	2.97^a^	2.32^b^	2.08^bc^	1.91bcd	1.65cde	1.47^de^	1.56^de^	1.42^e^	1.37^e^	1.44^e^	0.15	<0.001	<0.001	<0.001	<0.001
Mg (mg/kg)	442^a^	281^b^	223^bc^	208^cd^	178cde	167cde	163^de^	150^de^	142^e^	153^de^	20.7	<0.001	<0.001	<0.001	<0.001
Na (mg/kg)	886^a^	676^b^	603^b^	624^b^	609^b^	578^b^	589^b^	538^b^	507^b^	549^b^	62.3	<0.001	<0.001	<0.001	0.029
K (g/kg)	2.28^a^	2.00^ab^	1.98^ab^	1.94^ab^	1.83^ab^	1.79^b^	2.01^ab^	1.89^ab^	1.93^ab^	1.98^ab^	0.16	0.060	0.002	0.006	0.47
Cl (g/kg)	1.70^a^	1.06^b^	0.89^b^	1.12^b^	1.15^b^	1.08^b^	1.08^b^	1.07^b^	1.00^b^	1.15^b^	0.11	<0.001	<0.001	<0.001	0.82
S (g/kg)	2.21^a^	1.27^b^	0.83^c^	0.64^cd^	0.563^de^	0.49^de^	0.52^de^	0.46^e^	0.44^e^	0.45^e^		<0.001	<0.001	<0.001	<0.001
	(1.88–2.60)	(1.08–1.50)	(0.70–0.97)	(0.54–0.76)	(0.48–0.66)	(0.42–0.57)	(0.44–0.60)	(0.37–0.54)	(0.38–0.52)	(0.38–0.53)					
Zn (mg/kg)	33.9^a^	16.4^b^	10.5^c^	9.66^cd^	8.23^cd^	7.51^cd^	7.71^cd^	6.73^cd^	6.23^d^	6.97^cd^	1.27	<0.001	<0.001	<0.001	<0.001
Cu (μg/kg)	202^a^	157^b^	162^b^	148^b^	111^c^	92.1^c^	82.7^c^	80.9^c^	77.2^c^	92.0^c^	16.9	<0.001	<0.001	<0.001	<0.001
Mn (μg /kg)	24.3^a^	14.7^b^	8.64^c^	6.52^cd^	4.89^d^	4.84^d^	6.39^cd^	6.48^cd^	6.28^cd^	6.96^cd^		<0.001	<0.001	<0.001	0.48
	(16.4–36.1)	(9.89–21.8)	(5.83–12.8)	(4.39–9.70)	(3.30–7.24)	(3.29–7.13)	(4.34–9.40)	(4.40–9.53)	(4.27–9.24)	(4.67–10.4)					
Fe (mg/kg)	1.17^a^	0.74^b^	0.60^bc^	0.46^cd^	0.40cde	0.34^de^	0.35^de^	0.30^de^	0.26^e^	0.28^e^		<0.001	<0.001	<0.001	<0.001
	(0.92–1.49)	(0.58–0.95)	(0.47–0.76)	(0.36–0.59)	(0.31–0.51)	(0.27–0.42)	(0.28–0.44)	(0.24–0.38)	(0.21–0.33)	(0.22–0.36)					
Co (μg/kg)	3.19^a^	2.11^b^	1.37^c^	1.02^cd^	0.90^de^	0.73def	0.66^ef^	0.57^f^	0.58^f^	0.56^f^		<0.001	<0.001	<0.001	<0.001
	(2.60–3.91)	(1.71–2.59)	(1.12–1.68)	(0.83–1.26)	(0.73–1.10)	(0.60–0.89)	(0.55–0.81)	(0.47–0.70)	(0.48–0.70)	(0.47–0.71)					
Se (μg/kg)	294^a^	186^b^	120^c^	101^cd^	88.6cde	74.7^de^	77.2^de^	73.3^e^	72.3^e^	73.4^de^		<0.001	<0.001	<0.001	<0.001
	(251–345)	(157–219)	(102–140)	(86.1–120)	(75.6–104)	(64.3–86.7)	(66.5–89.7)	(63.1–85.1)	(62.2–84.0)	(62.7–86.0)					
Mo (μg/kg)	50.4^a^	30.2^b^	25.8^b^	25.4^b^	27.3^b^	25.5^b^	26.8^b^	28.4^b^	26.3^b^	26.3^b^	32.7	<0.001	<0.001	<0.001	0.93
Osmolality (mOsm/kg)	348^a^	315^b^	300^c^	292^cd^	288^de^	287^de^	286^de^	286^de^	281^e^	280^e^	3.9	<0.001	<0.001	<0.001	<0.001

a–f Means with different letters within rows are significantly different (*P* < 0.05).

1 CvT = colostrum (milking 1) vs. transition milk (milkings 2–6); CvM = colostrum (milking 1) vs. mature milk (milkings 8–14); TvM = transition (milkings 2–6) vs. mature milk (milkings 8–14).

2 Standard error of the difference. Values excluded for log-normal data.

Structural macrominerals displayed patterns consistent with their roles in skeletal development and their association with milk protein secretion. Calcium yield tended to increase over time (*P* = 0.067), whereas its concentration declined significantly from colostrum to mature milk (*P* < 0.001). A similar pattern was observed for P, with concentrations being the greatest in colostrum and decreasing progressively from transition to mature milk (*P* < 0.001), whereas the yield remained unchanged (*P* = 0.76). As Ca and P are structurally integrated within casein micelles and secreted with milk protein, our findings are consistent with established milk composition principles, where an increase in milk volume can offset declining concentrations, resulting in increased or maintained total transfer of these structural minerals (Walstra et al., 2005). Although both minerals are associated with casein micelles, Ca exists in multiple milk fractions, including soluble and casein-bound pools, whereas P is more tightly regulated within casein micelles, potentially explaining increasing Ca yield with rising milk volume. Thus, the decline of the structural mineral concentration likely reflects dilution during the increase of milk secretion, whereas a stable increase in yield indicates a sustained delivery of skeletal building blocks to the neonate.

The concentrations of Mg and S declined throughout the course of lactation (*P* < 0.001). Even though Mg contributes to the skeletal structure (Underwood and Suttle, 1999), its primary role is as a metabolic cofactor, and weak association with casein micelles (Walstra et al., 2005) suggests that this pattern reflects dilution with increasing milk secretion rather than altered regulation of Mg transfer. In contrast to Mg, S in milk is primarily present as a component of amino acids within proteins (NASEM, 2021). The decline in S over time likely reflects the decrease in total milk protein concentration from colostrum to mature milk, rather than regulation of S as a free, independent mineral nutrient. Consistent with this explanation, in our study the relative decrease in S (∼79%) closely reflected the reduction in milk protein (∼76%) across the transition from colostrum to mature milk. In comparison, Ca and P, also associated with milk proteins but more tightly integrated with casein micelles, declined to a lesser extent (∼41% and ∼51%, respectively), indicating that the temporal changes in S more closely reflect changes in milk protein, although P is not exclusively protein-associated and is also present in phospholipids.

Sodium, K, and Cl exhibited responses during early lactation. Sodium and Cl were the highest in colostrum and declined thereafter (*P* < 0.001), whereas K concentration was the highest in colostrum and did not change until milking 6 (*P* = 0.060; Table 2). Yields of Na, K, and Cl increased over time (*P* ≤ 0.012), reflecting an increase in milk volume. Given their roles in osmotic balance and acid-base regulation, these minerals are more appropriately interpreted based on concentration versus yield during early lactation. The observed increases in electrolyte yields likely reflect increased milk secretion and associated water transfer from the dam to the calf, rather than changes in active mammary secretion or regulation of these minerals. Similar electrolyte patterns have been observed during the transition from early lactation to established lactation (Ontsouka et al., 2003), a period characterized by changes in mammary epithelial cell tight junction integrity and milk secretory activity.

In addition, osmolality was highest in colostrum (348 ± 3.1 mOsm/kg) and declined through transition milk (296 ± 1.5 mOsm/kg) and mature milk (*P* < 0.001; 283 ± 1.4 mOsm/kg). Both colostrum and transition milk were at levels considered hypertonic, whereas mature milk was isotonic relative to previously reported plasma osmolality in calves (Wilms et al., 2019, 2020). Although milk is generally maintained near plasma osmolality (Wilms et al., 2019), the elevated osmolality observed in colostrum likely reflects its unique composition, with similar values reported for pooled maternal colostrum (330 mOsm/kg; Quigley et al., 2019). Lactose is the primary driver of osmotic water secretion into milk and accounts for more than half of milk osmolality (Holt and Jenness, 1984; Hamon et al., 2025). Consistent with this mechanism, lactose in the present study increased ∼72% from colostrum to mature milk, suggesting that lower lactose concentrations in colostrum limit osmotic water secretion (Fischer-Tlustos et al., 2020). This pattern aligns with the literature showing that lactose is the lowest in colostrum and increases during early lactation, often inversely related to other milk components such as protein and fat (Parish et al., 1950; Fischer-Tlustos et al., 2020). However, because milk remains near isotonic with plasma, reduced lactose-driven water secretion in colostrum is likely offset by higher concentrations of electrolytes such as Na, K, and Cl, contributing to the slightly elevated osmolality observed in early mammary secretions. Additionally, as described earlier, Na, K, and Cl concentrations were the highest in colostrum and decreased thereafter, and their elevated secretion before tight junction closure likely reflects increased paracellular movement into milk. Together with reduced lactose-driven water secretion, this results in a higher solute-to-water ratio and may partially explain the elevated osmolality observed in colostrum (Holt and Jenness, 1984; Hamon et al., 2025). Although protein concentrations are high in colostrum due to IgG, these large molecules likely contribute minimally to milk osmolality compared with small solutes such as lactose and electrolytes (Hamon et al., 2025). As lactation progresses and lactose increases, greater lactose-driven water secretion into milk dilutes these solutes, contributing to the decline in osmolality toward values typical of mature milk (Hamon et al., 2025). Whether this high osmolality observed in colostrum serves a physiological role or simply reflects the lack of osmotic pressure of lactose drawing water into the mammary gland remains unclear (Fox, 2009). Although moderately hypertonic solutions (<600 mOsm/L, ∼580 mOsm/kg in bovine milk) do not impair (Hildebrandt et al., 2020) and may even increase the rate of gastric emptying in calves (McGuirk, 2003), the functional significance of the elevated osmolality of colostrum for neonatal digestion has not been established.

Trace mineral (Zn, Cu, Mn, Fe, Co, Se, Mo) concentrations were also significantly higher in colostrum than transition and mature milk (*P* < 0.001). Relative comparisons indicated that Fe and Co concentrations had the greatest decline from colostrum to mature milk (∼81%), followed closely by Zn (∼80%), whereas Cu (∼59%) and Mo (∼47%) exhibited lower relative changes. Among these trace minerals, only Co yield declined from colostrum to mature milk (*P* = 0.032), whereas yields of Zn, Cu, Mn, and Fe were maintained or increased, suggesting that their transfer is sustained despite increasing milk production. Given that these trace minerals are tightly regulated, their yields likely reflect neonatal requirements for growth and metabolic function rather than passive changes in milk composition. On the contrary, Cu yield increased during early transition milk, peaking at milking 4 (*P* = 0.021) before returning to levels comparable to colostrum, suggesting that this brief increase may reflect short-term regulatory adjustments or neonatal requirements. The observed patterns in trace mineral yield may align with overall nutrient and energy transfer to support early growth. The high colostral concentration likely reflects direct transfer to support neonatal immunity, metabolic activation, and cellular differentiation after birth.

Our findings agree with the mineral concentrations in colostrum reported by Kehoe et al. (2007), although our values were higher than those reported in earlier studies (Sato and Murata, 1932a,b; Garrett and Overman, 1940; Kume and Tanabe, 1993; Kume et al., 1998; Tsioulpas et al., 2007). The mineral concentrations of transition and mature milk in the present study were higher than those reported by Kume and Tanabe (1993) and Tsioulpas et al. (2007); however, a similar temporal response was observed, with concentrations being highest in colostrum and decreasing over time. Garrett and Overman (1940) observed a similar temporal response, with the exception of K concentrations, which increased during the transition from colostrum to mature milk. Similar concentrations of Zn and Mn in transition and mature milk were previously reported by Sato and Murata (1932a,b). Lim et al. (2020), reported concentrations of select minerals in milk samples collected at d 3 of lactation; the concentrations of Ca, P, Mg, and K were similar to those reported herein. In contrast, the concentration of Zn in transition milk in the present study was roughly half that of the value reported by Lim et al. (2020), and the concentration of Fe was roughly 5 times lower. In mature milk, mineral concentrations were marginally greater than the values reported (Kume et al., 1998; Visentin et al., 2018).

Differences observed among studies likely reflect variation in breed, parity, diet, environmental conditions, and difference in analytical techniques used. Parity has been shown to affect the mineral concentration of colostrum, with greater parities having reduced concentrations of some minerals (Kume and Tanabe, 1993; Kume et al., 1998). Other factors, such as diet, seasonality, and the environment (e.g., heat stress), have been shown to affect mineral concentration in milk (Toharmat and Kume, 1997; Gaignon et al., 2018). These effects are unlikely to be consistent across all minerals, as their responses likely depend on their biological roles, with structural minerals more closely linked to milk production, osmotic minerals in fluid balance, and trace minerals supporting metabolic and enzymatic functions. It is also likely that differences in methods of quantification play some role in the lack of agreement of mineral concentrations across studies. Macrominerals were quantified using ICP-MS or ICP-OES, and the observed concentrations were comparable to those reported as Kehoe et al. (2007) and Lim et al. (2020), using similar analytical techniques. Although other studies have employed different methods of quantification and reported differing absolute values, the general temporal trends in mineral concentrations (i.e., decreasing during the transition from colostrum to mature milk) have been consistent across studies, except for K in the studies of Garrett and Overman (1940), and Kume et al. (1998).

Most previous studies did not report mineral yields, limiting direct comparisons. In the present study, changes of mineral yield were driven by an increase of milk volume and a decrease in mineral concentration across lactation. As a result, yields of macrominerals increased (K, Na, and Cl) or tended to increase (Ca) across lactation, whereas yields of some trace minerals showed opposing patterns, including a decline in Co and an increase in Cu. Both Kume and Tanabe (1993) and Kume et al. (1998) reported lower colostrum yields and mineral concentrations than observed in this study, which would result in lower mineral yields. These unique patterns suggest that yield data should be interpreted cautiously. For structural minerals, yield reflects total mammary secretion, and, as modern dairy cattle are selected for high milk production (Oltenacu and Broom, 2010), greater yield does not necessarily correspond to nutrient delivery to the calf; as such, concentration may be a more informative indicator compared with yield, especially in systems where milk intake is limited.

In summary, this dataset illustrates that temporal changes of minerals in milk in early lactation are highly specific to each mineral and its main role in milk. When maternal nutrient transfer leads as a factor, temporal mineral yields are mainly explained by this role. In contrast, when milk secretion or milk composition drives these changes, temporal milk concentrations describe this process. These results demonstrate that minerals in milk during early lactation can be interpreted by the mineral transfer to the calf (reflected by yield) or by the functional role of minerals within milk (reflected by concentration). Often, both processes could be at play, making interpretation difficult.

Most mineral concentrations were highest in colostrum and decreased in transition and mature milk. Following this transition, concentrations of most minerals remained relatively stable, suggesting a tight physiological regulation of mineral composition once lactation is established. In contrast, structural macrominerals exhibited patterns whereby total mineral yield increased with milk volume despite declining concentrations during early lactation. As modern dairy cows produce large volumes of milk that exceed the nutritional requirements of the calf, total mineral yield primarily reflects mammary secretion rather than direct nutrient delivery to the neonate. These findings can be used to understand the broader physiology in dairy cattle from prenatal supply in utero to postnatal delivery through colostrum and milk.
